# Audit of essential medicine listing and registration status of medicines on standard treatment guidelines in Kenya, Tanzania and Uganda: Case study of malaria, tuberculosis, hypertension and type 2 diabetes mellitus

**DOI:** 10.1177/20542704241232814

**Published:** 2024-03-29

**Authors:** Deborah Babatunde, Allyson M Pollock, Moses Ocan, Petra Brhlikova

**Affiliations:** 1 Health System Strengthening, APIN Public Health Initiatives, Abeokuta, Ogun State, Nigeria; 2 Population Health Sciences Institute, 5994 Newcastle University, Newcastle upon Tyne, UK; 3 Department of Pharmacology & Therapeutics, Makerere University, Kampala, Uganda

**Keywords:** essential medicines list, standard treatment guidelines, WHO treatment guidelines, drug register, malaria, tuberculosis, hypertension type 2 diabetes mellitus

## Abstract

**Objectives:**

To determine alignment between national and World Health Organization (WHO) treatment recommendations, medicines prioritisation in country's essential medicines list (EML), and medicines availability in National drug register.

**Design:**

An audit of medicines for malaria, tuberculosis, hypertension and type 2 diabetes mellitus listed in the national standard treatment guidelines (STGs) of Kenya, Tanzania and Uganda, as of March 2021, against WHO treatment guidelines, and respective country EML and National drug register.

**Setting:**

Not applicable.

**Participants:**

None.

**Main outcome measures:**

Proportion of medicine in country's STGs that align with WHO treatment recommendations, country's EML and country's drug register.

**Results:**

Some disease areas had two sets of treatment guidelines – national STGs and disease-specific treatment guidelines (DSGs) developed at different times with different recommended medicines. Both STGs and DSGs included medicines not recommended by the WHO or not listed on the country EML and drug register. Non-WHO-recommended medicines accounted for 17/68 (25%), 10/57 (18%) and 3/30 (10%) of all STG medicines in Kenya, Tanzania and Uganda, respectively. For tuberculosis, the numbers and proportion of STG medicines listed on the respective national EMLs were 2/6 (33%), 15/19 (79%) and 4/5 (80%) in Kenya, Tanzania and Uganda. All tuberculosis medicines included in Kenya's and Uganda's STGs were registered compared with only 12/19 (63%) tuberculosis medicines in Tanzania's STG.

**Conclusions:**

Alignment between treatment guidelines, EMLs and drug registers is crucial for effective national pharmaceutical policy. Research is needed to understand the inclusion of medicines on STGs and DSGs which fall outside WHO treatment guidelines; the non-alignment of some STGs and DSGs, and STGs and DSGs including medicines which are not on country EML and drug register.

## Introduction

The World Health Organisation (WHO) recommends that national standard treatment guidelines (STGs) are aligned with country essential medicines lists (EML) to promote appropriate prescribing and access to medicines.^1^ WHO has put in place a rigorous evidence-based approach for developing international disease-specific treatment guidelines (DSGs), and these usually form the basis for treatment guidelines in low- and middle-income countries.

National STGs are usually drawn up by national drug and therapeutics committees and ministries of health and draw on the WHO guidelines.^2^ Their target audience is healthcare providers, prescribers, managers and policy makers. Since national EMLs are used to guide medicines procurement for public health care in approximately 75% of low-income and 50% of middle-income countries,^3^ medicines recommended for use on a national STG should align with its country EML to ensure appropriate prescribing and use. However, STGs may not be updated when country EMLs are revised due to lack of capacity.

There is limited research into the alignment of medicines listed on STGs with country EMLs and drug registers, and no research into the extent to which country STGs align with WHO treatment recommendations. Robertson *et al.*^6^ found good alignment between Kenya's STGs and EMLs for selected medicines for children. Previous research has reported under-registration of some essential medicines, including antimicrobials and medicines for cryptococcal meningitis in Kenya, Tanzania and Uganda.^4,5^

The aim of this study is to compare medicines listed on national STGs against WHO treatment guidelines, respective country EML and National drug registers in Kenya, Tanzania and Uganda. We focus on medicines used in four conditions; malaria, tuberculosis, hypertension and type 2 diabetes mellitus as these are among the leading causes of death in these countries.^7–9^

## Methods

### Data sources (as of March 2021) and extraction

The data set was collected in March 2021.

WHO TGs: Malaria, TB, hypertension and type 2 diabetes mellitus treatment guidelines^10–14^ were retrieved from the WHO website (See Box 1).

Box 1. WHO, Kenyan, Tanzanian, and Ugandan treatment guidelines for malaria, tuberculosis, hypertension, and type 2 diabetes mellitus by date of issue and source.

**Table:** 

Data sources	WHO	Kenya		Tanzania		Uganda	
Treatment guidelines		STG	DSG	STG	DSG	STG	DSG
Malaria	2015^10^	2009^15^	2016	2017^17^	Not found	2016^16^	Not found
Tuberculosis	2017^14^, 2019^13^	2009^15^	2017^19^	2017^17^	2013,^26^ 2016,^27^ 2018^28^	2016^16^	2017^29^
Hypertension	2007^12^	2009^15^	2018^18^	2017^17^	Not found	2016^16^	Not found
Type 2 diabetes mellitus	2018^11^	2009^15^	2010^20^	2017^17^	Not found	2016^16^	Not found
EML	–	2019		2017		2016	
Drug register	–	2021		2021		2021	

STG, standard treatment guidelines; DSG, disease-specific guidelines.

Country STGs: In all three countries, STGs and EMLs are developed by national committees. All three countries each have a single national STG document that includes malaria, TB, hypertension and type 2 diabetes mellitus published on the websites of the respective ministries of health.^15–17^

DSGs: For all four diseases, Kenya has DSGs,^18–20^ which are more recent than the STGs (See Box 1).

Tanzania and Uganda have disease-specific guidelines for TB only (See Box 1). Tanzania also has DSGs of TB in children, which pre-date the national STGs, and guidelines for MDR-TB (See Box 1). The STGs and DSGs were also searched for funding sources, key references and contributing partners (See Appendix 1).

EMLs: National EMLs were downloaded from websites of the respective countries’ ministries of health; the 2019 version for Kenya, 2017 for Tanzania and 2016 for Uganda.^17,21,22^

National Drug Registers were accessed through the websites^23–25^ of the national drug authorities.

Medicines (name, dosage form and strength) for the four diseases were extracted from national STGs and DSGs, WHO treatment guidelines and National EMLs. Where medicine names were not provided, particularly in the WHO guidelines for hypertension and type 2 diabetes mellitus, the medicine classes were extracted. 19 medicines had no information on dosage forms and strengths in the three national STGs and DSGs and so were excluded as the comparison was mostly dosage form and strength specific.

Medicines listed on the WHO treatment guidelines for each disease were compared with those listed on national STGs and DSGs. In addition, medicines recommended in national STGs and DSGs were checked against respective country EMLs and drug registers^23–25^ to determine whether they were prioritised for public procurement and registered for use.

### Analysis and results

Table 1 shows the number of medicines on National STGs and DSGs which correspond to WHO treatment guidelines. Country STGs and DSGs listed only some of the WHO recommended medicines for the selected diseases except for hypertension for which Kenya's STGs included medicines from all classes recommended by the WHO.

**Table 1.** Number and proportion of WHO-recommended medicines listed on national STGs and in disease-specific guidelines (DSGs) by country and disease area.

WHO treatment guidelines (Publication Year)	Number of medicines recommended in the WHO guidelines	WHO-recommended medicines listed in national STGs, n (%)			WHO-recommended medicines listed in DSGs, n (%)		
		Kenya	Tanzania	Uganda	Kenya	Tanzania	Uganda
Malaria (2015)	14	5 (35.71)	6 (42.86)	9 (64.29)	8 (57.14)	-	-
Tuberculosis (2017)	19	5 (26.32)	18 (94.74)	5 (26.32)	18 (94.74)	17 (89.47)	17 (89.47)
Hypertension^a^ (2007)	8	8 (100.00)	6 (75.00)	6 (75.00)	7 (87.50)	-	-
Type 2 diabetes mellitus^a^ (2018)	6	3 (50.00)	4 (66.67)	3 (50.00)	4 (66.67)	-	-

a The treatment recommendations in WHO treatment guidelines were provided as medicine classes rather than specific medicine names.

Of the 19 medicines listed on the WHO treatment guidelines for tuberculosis, Kenya listed 5 and 18 on its STG and DSG, respectively; Tanzania listed 18 and 17, respectively and Uganda listed 5 and 17, respectively (Table 1). WHO-recommended medicines for multidrug-resistant tuberculosis (MDR-TB), including Bedaquiline and Delamanid were not included in Kenya's and Uganda's STGs. For malaria, less than two-thirds of the WHO-listed medicines were included on the national STGs for Kenya, Tanzania and Uganda and on the DSG in Kenya.

Table 2 shows medicines listed on national STGs which were not on WHO treatment guidelines. In Kenya, DSGs listed almost twice as many medicines for malaria as the STGs and four times as many medicines for tuberculosis. Some of these medicines fell outside WHO's recommendations.

**Table 2.** Number and proportion (%) of medicines on STGs and DSGs not listed on WHO treatment guidelines.

Diseases	Kenya	Tanzania	Uganda
Malaria			
Number of medicines in STG	8	8	9
Outside the WHO's recommendations	1 (12.50)	0 (0.00)	0 (0.00)
Number of medicines in DSG	14	-	-
Outside the WHO's recommendations	5 (35.70)	-	-
Tuberculosis			
Number of medicines in STG	6	19	5
Outside the WHO's recommendations	2 (33.33)	2 (10.53)	0 (0.00)
Number of medicines in DSG	25	22	28
Outside the WHO's recommendations	4 (16.00)	1 (4.55)	9 (32.14)
Hypertension			
Number of medicines in STG	42	24	11
Outside the WHO's recommendations	12 (28.57)	8 (33.33)	3 (27.27)
Number of medicines in DSG	32	-	-
Outside the WHO's recommendations	5 (15.63)	-	-
Type 2 diabetes mellitus			
Number of medicines in STG	12	6	5
Outside the WHO's recommendations	2 (16.67)	0 (0.00)	0 (0.00)
Number of medicines in DSG	12	-	-
Outside the WHO's recommendations	2 (16.67)	-	-

Tanzania's and Uganda's diabetes and malaria guidelines showed greater alignment with WHO recommendations than those for tuberculosis and hypertension. Uganda listed 9 (of 28) TB medicines on its DSG outside of WHO's recommendations while the STG for hypertension listed 3 (of 11). Tanzania's STGs included 8 (of 24) medicines for hypertension and 2 (of 19) medicines for tuberculosis that were not included in the WHO treatment guidelines.

Across the four diseases, the STGs in Kenya, Tanzania and Uganda listed a total of 17, 10 and 3 medicines, respectively, outside WHO recommendations. Of these, 7 (41.2%), 9 (90%) and 3 (100%) in Kenya, Tanzania and Uganda, respectively, were on the respective national EMLs.

Table 3 shows the number and proportion of medicines recommended in national STGs and DSGs by EML and registration status.

**Table 3.** Number and proportion (%) of medicines on STGs and DSGs listed on EMLs and on drug registers in Kenya, Tanzania and Uganda.

Diseases	Kenya	Tanzania	Uganda
Malaria			
Medicines in STG n (%)	8	8	9
Listed on EML	7 (87.50)	7 (87.50)	9 (100.00)
With equivalent registered products	7 (87.50)	8 (100.00)	9 (100.00)
Medicines in DSG n (%)	14	-	-
Listed on EML	13 (92.86)	-	-
With equivalent registered products	12 (85.71)	-	-
Tuberculosis			
Medicines in STG n (%)	6	19	5
Listed on EML	2 (33.33)	15 (78.95)	4 (80.00)
With equivalent registered products	6 (100.00)	12 (63.16)	5 (100.00)
Medicines in DSG n (%)	25	22	28
Listed on EML	17 (68.00)	17 (77.27)	22 (78.57)
With equivalent registered products	20 (80.00)	19 (86.36)	21 (75.00)
Hypertension			
Medicines in STG n (%)	42	24	11
Listed on EML	18 (42.86)	21 (87.50)	11 (100.0)
With equivalent registered products	25 (59.52)	14 (58.33)	10 (90.91)
Medicines in DSG n (%)	32	-	-
Listed on EML	15 (46.88)	-	-
With equivalent registered products	24 (75.00)	-	-
Type 2 diabetes mellitus			
Medicines in STG n (%)	12	6	5
Listed on EML	3 (25.00)	6 (100.00)	5 (100.00)
With equivalent registered products	6 (50.00)	5 (83.33)	5 (100.00)
Medicines in DSG n (%)	12	-	-
Listed on EML	5 (41.67)	-	-
With equivalent registered products	10 (83.33)	-	-

There is considerable variation across the countries in the number of medicines listed on guidelines for TB, hypertension, and diabetes, included on the EML and registered for use. There was much less concordance between treatment guidelines and EMLs and drug registers in Kenya compared with Uganda and Tanzania. Kenya had the highest number of medicines listed in treatment guidelines and the lowest proportion of those medicines listed on its EML. It also had a high number of recommended medicines without any registered products: 6/12 STG-recommended medicines for type 2 diabetes mellitus and 17/42 for hypertension had no registered products.

Uganda had the lowest number of medicines on its treatment guidelines and the highest proportion of medicines both on the EML and drug register. In Tanzania, all STG-recommended medicines for malaria were registered and all those for diabetes were listed on the EML.

## Discussion

The co-existence of national STGs and DSGs developed in different time periods with different treatment recommendations for the same disease areas has not previously been reported in the literature. National STGs are developed for use at all levels of healthcare in a country, including primary health facilities. DSGs do not specify healthcare levels but are intended for use by healthcare workers managing the disease (Appendix 1). The co-existence of outdated national STGs with more current disease-specific guidelines may give rise to inconsistency in the quality of healthcare delivered, particularly where guidelines differ in terms of treatment recommendations and their use. For instance, the omission of most WHO-recommended MDR-TB medicines from Kenya's and Uganda's STGs may result in inappropriate prescribing practices. This is particularly of concern in Kenya, which has a high burden of MDR-TB.^30^ It should also be noted that poly-pharmacy has been associated with the use of disease-specific guidelines to manage patients with multimorbidity.^31^

### Non-alignment of national treatment guidelines with WHO treatment guidelines, country EML and drug registers

Although not all medicines on WHO treatment guidelines are prioritised for use on national treatment guidelines, it was surprising to find a high proportion of medicines on national STGs not listed on WHO treatment guidelines. For example, although Kanamycin and Capreomycin are no longer recommended by WHO (2019) for the treatment of MDR-TB, they remain recommended on the 2017 Tanzanian STG.

The EML is the basis for government procurement and hence availability. However, some medicines listed on STGs in Kenya, Tanzania, and Uganda are not included on their respective country EML. Ideally, the national STGs should be revised periodically (every second year) and in line with the EML revisions.^1^ In reality, few countries have resources to achieve this. The first edition of the Ugandan STGs was developed in 2003, with revised editions in 2010, 2012 and 2016, and in 2023 undertaken in line with the Ugandan EML. In Tanzania, EML and STG revisions are aligned, with versions published in 1991, 2007, 2012, 2013, 2017 and 2021. Kenya does not update its STG in conjunction with the EML; its STGs were last updated in 2009 and its EML in 2019. Our study showed greater concordance between STGs and EMLs in Tanzania and Uganda where efforts are being made to align the revision of both policy documents. However, despite the coordination of STG and EML revisions in Tanzania, our analysis revealed that not all STG-recommended medicines were listed on the EML in 2017. Further studies should seek to assess the concordance between STG, EML and the availability of medicines.

While there is great variation in the use of national guidelines by health care workers between countries, their importance in prescribing is evident from studies of health care workers adherence to them. For instance, some 63.1% and 54.6% of healthcare workers adhere to malaria DSGs in Uganda and Tanzania respectively.^32,33^ In Uganda, only half of prescriptions assessed complied with national STGs and adherence to guidelines largely depended on the availability of recommended medicines.^34^ Around a third (29.9%) of healthcare workers in Tanzania adhere to national treatment guidelines.^35^ Similar findings have been reported for South Africa where 56.6% and 63.6% of doctors and nurses, respectively, adhere to national treatment guidelines for hypertension.^36^

In general, essential medicines are more available and affordable than non-essential medicines in both private and public sectors.^37,38^ Where medicines are prescribed outside the EML, they may be available in the private sector, particularly in community pharmacies and patent medicines stores where research has shown that private facilities sell medicines to patients at 9–20 times the international reference prices.^39–41^ Our findings support previous recommendations that STG-recommended medicines should be aligned with EMLs to reduce and control medicine prices and improve medicine access and availability.^42^

Of concern is the high proportion of medicines listed on national treatment guidelines that are not registered for use –some of these include essential medicines. Previous research found that 30% of antiretrovirals circulating on the market in Kenya and 14% of antimalarials in six African countries were unregistered.^43,44^ Although availability of unregistered products could be due to the use of special import licence to supply medicines,^5^ the other concern is the circulation of falsified and substandard medicines in the absence of medicine registration. Registration of medicines recommended in treatment guidelines will not only ensure that medicines for priority health conditions in a country are locally available but will also ensure that regulatory efforts focus on priority medicines.

Other factors influencing access and appropriate prescribing and use are cost, prescribers’ adherence to guidelines and EMLs and patient adherence.

## Conclusion and recommendations

The co-existence of parallel national STGs and disease-specific guidelines developed at different times, and with different treatment recommendations is a new finding that needs further research to establish the basis of their use. All treatment guidelines should be updated, harmonised and aligned with country EMLs and WHO treatment guidelines. Countries should prioritise registration of essential medicines and monitor and evaluate adherence to treatment guidelines and EMLs to promote medicines availability and appropriate use. Future research should also analyse prescribing practices against treatment guidelines, the country EML and drug register. Qualitative research is also needed to understand the development of alternative guidelines to STGs and their application and use within the country.

## Appendix 1

Kenyan, Tanzanian and Ugandan treatment guidelines for malaria, tuberculosis, hypertension and type 2 diabetes mellitus; publisher and publication year, target group, Funding Support, Key References, Acknowledged Developing Partners who facilitated/supported the development of the guidelines.

**Table:**

	Kenya	Tanzania	Uganda
National standard treatment guidelines			
Publisher, year	Ministry of Medical services and Ministry of Public Health and Sanitation (unified under Ministry of Health since 2013), 2009^15^	Ministry for Health, Community Development, Gender, Elderly and Children, 2017^17^	Ministry of Health, 2016^16^
Target audience	All health professionals working in clinical settings	Prescribers and Dispensers	Health workers and Health Managers
Funding support	European Commission/African Caribbean Pacific/ WHO partnership (EC/ACP/WHO)	PATH under Access Delivery Partnership in collaboration with PRICELESS South Africa. Swiss Agency for Development and Cooperation (SDC), Global Fund for AIDS, TB and HIV.	USAID-funded Uganda Health Supply Chain (UHSC), and the Clinton Health Access Initiative (CHAI).
Acknowledged partners	WHO	PATH under Access Delivery Partnership in collaboration with PRICELESS South Africa. Swiss Agency for Development and Cooperation (SDC), Global Fund for AIDS, TB and HIV.	USAID-funded Uganda Health Supply Chain (UHSC), and the Clinton Health Access Initiative (CHAI), WHO
Key references	Not specified	*Achan J et al. (NEJM 2012) ‘Antiretroviral agents and prevention of malaria in HIV infected Ugandan Children’*	WHO 2015. *Guidelines for the treatment of Malaria* Medecins Sans Frontieres 2016. *Clinical Guidelines – Diagnosis and treatment manual* Rep. of South Africa. Essential Drugs Programme. *Hospital (Paediatrics) Standard Treatment Guidelines and Essential Medicines List.* 3rd ed. Rep. of Namibia. Ministry of Health and Social Services, 2011. *Namibia Standard Treatment Guidelines.* BMJ Group and the Royal Pharmaceutical Society of Great Britain, 2014. *British National Formulary* Medscape. http://www.medscape.com
Malaria Disease Guidelines			
Publisher, year	Ministry of Health, 2016	Not found	Not found
Target audience	All health professionals		
Funding support	USAID PMI, UK DFID		
Acknowledged partners	WHO, Global Malaria Program		
Key references	WHO 2015. Guidelines for the treatment of malaria		
Tuberculosis Disease Guidelines			
Publisher, year	The Ministry of Health and the National Tuberculosis, Leprosy and Lung Disease program (NTLD-P), 2017^19^	Ministry of Health and Social Welfare National Tuberculosis and Leprosy Programme, 2013,^26^ 2016,^27^ 2018^28^	Ministry of Health under the Uganda National Tuberculosis and Leprosy Control Programme, 2017^29^
Target audience	All health workers involved in TB care	Health workers and other stakeholders	All involved in TB and Leprosy prevention and care in Uganda
Funding support	Not specified	Global Fund ATM, USAID, HIDN	Not specified
Acknowledged partners	CDC, KAPTLD, NASCOP, Moi University (AMPATH), University of Nairobi, Counties, WHO, CHS, MSF, HSO, Stop TB Partnership, PATH, MSH, KNH and NTLD-P Staff	NTLP Staff, MSH, PATH, German Leprosy and TB Relief Association, Regional and Council Health Management Teams, Regional and District Tuberculosis and Leprosy Coordinators, and TB/HIV Officers	USAID (TRACK-TB Project), Uganda Health Supply Chain, CHAI, Representatives from Makerere University College of Health Sciences, German Leprosy Relief Association
Key references	WHO 2006 Guidelines for the programmatic management of drug-resistant tuberculosis WHO 2003 Treatment of tuberculosis: Guidelines for national programs	WHO (2016). WHO treatment guidelines for drug-resistant TB WHO 2018. WHO treatment guidelines for isoniazid-resistant TB WHO 2012. Global Tuberculosis Report 2012	WHO 2015. Global tuberculosis report 2015. WHO 2015. Guidelines on the management of latent tuberculosis infection. WHO 2010. Treatment of tuberculosis: guidelines. CDC 2003. Treatment of tuberculosis.
Hypertension Disease Guidelines			
Publisher, year	The Division of Non-Communicable Diseases - Ministry of Health, 2018^18^	Not found	Not found
Target audience	All health workers and all health institutions		
Funding support	Healthy Heart Africa project, WHO Kenya Country Office and AIHD		
Acknowledged partners	Kenya Cardiac Society, Kenya Pediatric Association, University of Nairobi, AMPATH, Kenyatta University, NASCOP, Unit of Specialized Services (MOH), AMREF, CHAK, MSF-Belgium, Kenya Society of Thrombosis and Hemostasis, Kenyatta National Hospital and NCD Alliance-Kenya		
Key references	Gabb GM *et al.* (Mortality 2016) ‘Guideline for the diagnosis and management of hypertension in adults – 2016 Seedat Y *et al.* (SAJDVD 2014). South African hypertension practice guideline 2014		
Type 2 Diabetes Mellitus Disease Guidelines			
Publisher, year	Ministry of Health in collaboration with Non-Governmental Organizations, Regional and International Diabetes Support Bodies, 2010^20^	Not found	Not found
Target audience	Health workers		
Funding support	Ministry of Public Health and Sanitation, World Diabetes Foundation, International Diabetes Federation		
Acknowledged partners	World Diabetes Foundation, International Diabetes Federation, Diabetes Management Information Centre, University of Nairobi and Kenyatta National Hospital, Diabetes Kenya Association		
Key references	Canadian Diabetes Association 2003 Clinical Practice Guidelines for the Prevention and Management of Diabetes in Canada (CAN J DIABETES 2003) International Diabetes Federation, Western Pacific Region (WPR) 2001 ‘Type 2 Diabetes Practical Targets and Treatments Guidelines’ American Diabetes Association: Clinical Practice Recommendations 2003. (Diabetes Care 2003)		
